# Application of Teledermoscopy in the Diagnosis of Pigmented Lesions

**DOI:** 10.1155/2018/1624073

**Published:** 2018-10-10

**Authors:** C. B. Barcaui, P. M. O. Lima

**Affiliations:** ^1^Adjunct Professor of Dermatology, Faculty of Medical Sciences, State University of Rio de Janeiro, PhD in Medicine (Dermatology), by University of São Paulo, Dermatology Department, Pedro Ernesto University Hospital, Rio de Janeiro State University, Rio de janeiro, Brazil; ^2^Physician Residing in Dermatology, Department of Dermatology, Pedro Ernesto University Hospital, State University of Rio de Janeiro, Rio de Janeiro, Brazil

## Abstract

**Background:**

Dermatology, due to the peculiar characteristic of visual diagnosis, is suitable for the application of modern telemedicine techniques, such as mobile teledermoscopy.

**Objectives:**

To evaluate the feasibility and reliability of the technique for the diagnosis of pigmented lesions.

**Methods:**

Through the storage and routing method, 41 pigmented lesions were analyzed. After the selection of the lesions during the outpatient visit, the clinical and dermatoscopic images were obtained by the resident physician through the cellphone camera and sent to the assistant dermatologist by means of an application for exchange of messages between mobile platforms. Firstly, the assistant dermatologist described the visualized dermatoscopic structures and defined its diagnosis and conduct, based solely on the evaluation of the clinical and dermatoscopic images, without having the knowledge of the anamnesis data. Afterwards, the same assistant dermatologist evaluated the patient face to face, defining the dermatoscopic structures, diagnosis, and conduct. The data obtained through teledermoscopy and face-to-face assessments were compared and accuracy was defined as the concordance between the diagnoses.

**Results:**

A match rate of 90% between teledermoscopic and face-to-face diagnosis was demonstrated (McNemar's statistical analysis, whose p value was 0.1366, showed no evidence to support the inferiority of the teledermoscopic method).

## 1. Introduction

The reduction of the morbidity and mortality of nonmelanoma skin cancer and melanoma is the greatest current challenge for dermatology and, within this context, this includes the early diagnosis of melanoma, dermatoscopy, teledermatology, and teledermoscopy.

Early diagnosis results in a better prognosis for the patient (thin, <1mm, nonulcerated melanomas have a 95% survival rate within 5 years, whereas Breslow ulcerated melanomas> 4mm and lymph node metastasis have only 24% survival at 5 years), and dermoscopy is essential, since it consists of a more accurate method for the diagnosis of melanoma than the naked eye one, increasing the detection of early-stage melanoma by up to 49% [1].

Dermatology, due to the peculiar characteristic of visual diagnosis, is ideal for the application of modern telemedicine techniques, with several recent studies proving the viability and reliability of teledermatology and, in particular, teledermoscopy, with high levels of concordance in diagnosis and management plan in relation to face-to-face consultation [2].

The World Health Organization defines telemedicine as the use of health communication technologies for the exchange of medical information for diagnosis, treatment, prevention, research, evaluation, and education. One of the existent ways of telemedicine is teledermatology, which is already well established, whose publications began in 1995 and these ones have been growing exponentially. Within teledermatology, teledermoscopy appears as a promising area for the diagnosis and management of pigmented skin lesions, early detection of skin cancer, and screening [2].

Teledermatology has two distinct operation models, the synchronous, through videoconference and satellite communication, which occurs in real time and the asynchronous, through a storage and routing system, including the use of e-mail, web, and mobile teledermatology, and which provides high levels of diagnostic accuracy, with lower cost, greater convenience, and practicality [3].

Storage and routing teledermatology are constantly growing around the world with improvements in communication and imaging technologies, allowing expert judgment in situations in which access to a dermatologist might be difficult due to geographic distance or excessive demand.

Mobile teledermoscopy consists of a new application of teledermatology, in which clinical and dermatoscopic images are captured and transmitted by mobile devices (e.g., smartphones, tablets) [4]. The image quality of these devices has been improved and no longer represents a barrier in teledermatology [5].

In this mobile teledermoscopy study, the first one developed in Brazil, we aimed to study the feasibility and reliability of the technique for the dermatological diagnosis of pigmented lesions.

## 2. Methods 

Patients were prospectively selected from the outpatient clinic of the Department of Dermatology from April to June 2017. The inclusion criteria consisted of men or women, of any age, with pigmented lesions, whether melanocytic or not. After the selection of the lesions during the outpatient visit, the clinical and dermatoscopic images were obtained by the resident physician and sent to the assistant dermatologist before face-to-face assessment.

The clinical images were obtained using the cell phone camera (Iphone 6 model A1549, with an integrated camera of 8 megapixels, resolution 3264x2448 pixel, digital stabilization, autofocus and without flash, with a good natural lighting) in two panoramic and macromodes (at an established distance of 20 cm from the lesion to be further studied). The dermatoscope which has been used was DermLite DL4 from 3Gen®, San Juan Capistrano, CA 92675, USA; and, for the acquisition of the dermatoscopic images, the camera lens was applied to the DermoLite® MagnetiConnect TM device of the 3Gen® Connection Kit for iPhone6 P / N: DLCKi6-MC, San Juan Capistrano, CA 92675, USA, with the dermatoscope at position 0, in polarized mode, without making use of flash or zoom camera assets. To the large lesions, we performed more than one dermatoscopic image.

At first, the images were sent via WhatsApp Messenger, a free messaging application available for the iPhone and other platforms, to the assistant dermatologist with extensive experience in dermatoscopy, which described the dermatoscopic structures visualized, as well as the analysis of patterns, when these ones were applicable; and the diagnosis and procedures were defined, based only on the evaluation of the clinical and dermatoscopic images, without the knowledge of the anamnesis data.

At the second moment, the same assistant dermatologist evaluated the patient face-to-face, using the same dermatoscope and having access to anamnesis data (sex, age, location and time of the injury evolution, local symptoms, personal or family history of melanoma, comorbidities, and relevant aspects of the anamnesis), defining the dermatoscopic structures and / or pattern analysis, diagnosis, and procedures.

The data obtained through teledermoscopy and face-to-face examinations were compared, the differences between them being analyzed and what was essential being defined, in face-to-face examination, for decision-making. The face-to-face examination was defined as the gold standard for the final procedure. The diagnostic accuracy was defined as the agreement between the teledermoscopic diagnosis and the gold standard. Clinical and dermatoscopic face-to-face diagnoses were considered suitable in patients with clinically and teledermoscopically benign and nonsuspected lesions and no biopsy procedure was performed. The lesions considered malignant or suspicious were studied histopathologically and were correlated with clinical-dermatoscopic diagnosis.

The diagnostic agreement between teledermatology/teledermoscopy and face-to-face assessment was calculated using the McNemar test, hypothesis test for paired data. This test works with two hypotheses: the null hypothesis, in which there is no difference between the representativeness between the diagnoses by the two methods and the alternative hypothesis, in which there is statistical difference. At the significance level of 5% (0.05), the null hypothesis is accepted when the p value is greater than 0.05.

## 3. Results 

Forty-one lesions were studied in 31 patients, 22 females, and 9 males. The mean age was 56.5 years (11 to 78 years). The lesions were present for an average duration of 80.4 months (from 1 day to 40 years), excluding those not specifically determined, only reported as present ones since childhood or as time of unknown onset. The specific locations included back (11 lesions), hand (7 including palmar lesion), malar (4), upper eyelid (2), cervical (2), thorax (2), breast (2), thigh, plantar (2), infraorbital (1), temporal (1), nose (1), retroauricular (1), armpit (1), abdomen (1), and leg (1 lesion). Eight patients reported local symptoms in the lesions, which consisted of growth (4), bleeding (1), color change (2), and pruritus (1). No patient had previous history of melanoma, one of whom had a personal history of basal cell carcinoma and one that had a family history of melanoma (first degree relative). Nineteen patients reported comorbidities: psoriasis (3), systemic arterial hypertension (8), diabetes (3), bullous lupus (1), cystic fibrosis (1), renal transplantation (1), and Sjogren's syndrome (1). We highlight other relevant aspects in the anamnesis in 10 cases, which consisted of the use of immunosuppressants (2), immunobiological (1), smoking (1), outdoor work profession (1), contact with exogenous pigment (1), inflammatory pigmentation of the underlying disease (1), application of acid to the lesion (1), and nonapplication of acid to the lesion (1).

From the clinical images obtained, two were reported by the teledermatologist as not perfectly made ones in focus and in one of the other cases there was an equivocal perception of the lesion size. All the dermatoscopic images were considered excellent.

There was no injury to the teleanalysis of the lesions in any of the cases. Teledermoscopic diagnoses and those established after face-to-face analysis are shown in Table 1 (lesions 1 to 41).

The teledermoscopic diagnoses were pigmented basal cell carcinoma (3), intradermal nevus (6), seborrheic keratosis (4), benign melanocytic lesion (12), blue nevus (3), intracorneal hematoma (1), atypical nevus melanocytic suspicion (2), dermatofibroma (1), congenital nevus (1), solar lentigo (2), and melanoma (4).

On the other hand, the face-to-face diagnoses consisted of pigmented basal cell carcinoma (4), intradermal nevus (6), seborrheic keratosis (4), benign melanocytic lesion (14), blue nevus (3), exogenous pigmentation atypical nevus (2), dermatofibroma (1), congenital nevus (1), solar lentigo (2), and melanoma (3).

The anatomopathological study was performed on 8 lesions and 4 pigmented basal cell carcinomas (lesions 1, 18, 25, and 35), 1 junctional melanocytic nevus (lesion 21), and 3 melanomas (lesions 29, 32, and 40) were found.

The agreement between the evaluations was 90%, and this reduction of 10 percentage points was not considered statistically significant, once there was no difference between the representativeness of the cases, since the p value was 0.1366. In other words, even though there is a reduction in concordance for teledermoscopy, there is insufficient statistical evidence to prove that this method is inferior, which can be considered equivalent to the traditional method (face-to-face assessment).

Discordant results included exogenous pigmentation, a pigmented basal cell carcinoma, and two benign melanocytic nevi.

The exogenous pigment lesion (lesion 9) was present 1 day ago in the right second finger of a 57-year-old female patient with a family history of melanoma and consisted of a discontinuous brownish macula, with poorly defined limits and linear medial aspect of the proximal interphalangeal joint to the dorsal distal interphalangeal joint. Dermoscopy revealed spots and globules, some brownish and other blackish ones, in the center and periphery, irregularly distributed along the lesion, with areas without dermatoscopic structures. The teledermoscopic diagnosis was of intracorneal hematoma. In the anamnesis, the contact with synthetic paint was revealed and the diagnosis was of exogenous pigmentation. In this case, the determining factor for the decision making was the data obtained during the anamnesis, from contact with the exogenous pigment. The conduct was expectant.

One case of benign melanocytic nevus was a 66-year-old man with a diagnosis of bullous lupus, with a dark brown macula on the back, for an unknown time, with no local symptoms, and with no melanoma history (lesion 16). In the dermoscopy analysis, reticulum-globular pattern, irregular net, and globules were identified with the colors light brown, dark brown, and gray. The diagnosis was of suspected malignant melanocytic lesion and the decision-making to be taken would be the excisional biopsy. In face-to-face assessment, the knowledge of the underlying disease and its pathophysiology (damage to basal epidermal cells), as well as the presence of suggestive lesions of disease activity, has modified diagnosis and decision-making for benign melanocytic lesion with inflammatory pigmentation of the disease and clinical follow-up.

The third discordant case (lesion 25) was referred to the male patient, 62 years old, renal transplant, using immunosuppressants, with lumbar lesion for unknown time, whose telediagnostic conclusion was melanoma or recurrent nevi (multicomponent pattern; presence of irregular mesh, stretch marks, spots, erythema, and blue-gray veil). The face-to-face assessment concluded that this one was pigmented basal cell carcinoma, where arboriform telangiectasia at the periphery of the lesion and leaf structures as well are identified. The anatomopathological study confirmed the diagnosis established in the face-to-face assessment.

Finally, the other case of benign melanocytic nevus was a 61-year-old female patient, who had a pigmented lesion in the dorsal median line, for unknown time, with atypical pigmentary network teledermoscopy and striae (lesion 39). In the face-to-face assessment, the dermoscopy was reassuring, in which a homogeneous pigment network was visualized. In this case, it was decided to perform the digital dermatoscopy, which showed a similar image to that of the teledermoscopy one. We suppose that the location of the lesion was the difficult point in the teledermoscopic and digital analysis, due to the difficulty in coupling the dermatoscope in maintaining the focus on the images capture.

There was one case where the palpation of the lesion was essential for the diagnostic conclusion, referring to the lesion 23, in which the telediagnostic hypotheses were of solar lentigo associated with blue nevus or melanocytic lesion with eccentric homogeneous pigmentation.

We also emphasize the case of lesion 21, which concerned a 22-year-old female patient, with a cystic fibrosis diagnosis, presenting a hyperpigmented macule, blackened in the center, and dark brown in the edges, present for 2 years, which evolved with the darkening of the lesion. The teledermoscopy identified an irregular network, striae, and gray-blue areas. The diagnostic hypotheses were atypical melanoma or melanocytic nevus and excisional biopsy as the conduct to be performed. In the face-to-face assessment, the same dermatoscopic structures were identified, maintaining the same dermatoscopic description, the same hypotheses, and the same conduct; however, the assistant dermatologist reported a more reassuring aspect after a global analysis of the case. The anatomopathological study revealed junctional melanocytic nevus associated with marked degree of melanin pigment incontinence

## 4. Discussion 

The concern with confidence in the diagnosis of skin malignancy with teledermatology is reflected in the current orientation that all suspicion of malignancy of the skin should be seen face to face [6]. However, the incorporation of high-quality teledermoscopic images in addition to macroscopic images may challenge this premise. The comparisons showed that the face-to-face and teledermoscopic correlation of the pigmented lesions is high.

In 1999, the teledermoscopic study carried out by Piccolo et al. (Italy and Austria) analyzed pigmented lesions of 66 patients in two groups (face-to-face and asynchronous teledermoscopy) and found agreement in 60 cases (91%) [7]. All lesions were histopathologic and concordance between face-to-face diagnosis was 92% and 86% for teledermoscopy [7]. Although the number of correct diagnoses were lower in teledermoscopy, it was not statistically significant [7]. The 6 discordant cases between face-to-face and teledermoscopy were classified as high (4) and medium (2) degree of difficulty [7].

Massone et al., in Austria in 2008, carried out the first teledermoscopy study using cellular phones for image capture and the storage and referral system for two teledermoscopists, in which diagnostic correspondence was found between the face-to-face examination of pigmented lesions in 89% and 94 % [8]. In 2011, Kroemer also used cell phones to capture clinical and dermatoscopic images without a special mobile pocket dermatoscope adapter and analyzed clinical and dermatoscopic images of 80 patients (104 lesions) separately, finding a concordance of 85% and 79%, respectively, compared to face and /or anatomopathological one; the value of the clinical image evaluation combined with clinical information in the diagnosis of skin tumors was attributed as relevant [9]. A total of 322 clinical images and 278 dermatoscopic images were obtained, of which 1% and 6% were considered inadequate for decision-making. The quality reduction of the dermatoscopic image was grounded by the lack of a special dermatoscope mobile phone adapter [9].

In 2016, Arzberger et al. showed excellent agreement on recommendations (“self-monitoring”, “short-term monitoring”, and “excision”) between dermatologists (face-to-face and teledermatology) in a cohort of 70 patients with moderate to high risk [1]. However, also in 2016, the study of dubious melanocytic lesions published by de Giorgi et al. highlighted the limitations of teledermoscopy when analyzing 10 challenging pigmented lesions by 10 different teledermatologists and demonstrated that the diagnostic concordance of the telediagnosis decreased after the observation of the (kappa statistical analysis between the histopathological diagnosis: face-to-face 0.6, clinical teledermatology 0.52 and teledermoscopy 0.38), which was justified by the complexity and dermatoscopic difficulty of the selected cases, including Spitzoid proliferation and atypical melanocytic nevus of the elderly, which can represent a pathological and a potential diagnostic failure due to its confusing dermatoscopic characteristics [1].

Another teledermoscopic challenge is the diagnosis of hypo- or nonpigmented lesions, as demonstrated by Fabbrocini in the study of pink-lesions (poor or absent pigmentation, absence of a regular network, and diameter less than 5mm), in which the clinical and dermatoscopic telediagnosis showed less diagnostic accuracy than the face-to-face one [1, 2]. In this same study, the dermoscopic structures were evaluated as for the best visualization, whether face-to-face or in dermatoscopic imaging [1, 2]. The conclusion drawn from this was that leaf structures, pseudocysts, comedo-like openings, “blue-white structures”, and “blotches” are detected with the same frequency in face-to-face analysis and in teledermoscopy [1, 2]. Other structures such as pigment network, regression structures, and diffuse pigmentation are more evident in teledermoscopic observation, whereas the vascular pattern, radiated streaks, and spots/globules are less frequently detected [1, 2].

An important factor that should be considered is the standardization of image and service equipment. There are still no universal imaging standards developed and implemented in teledermatology; however, there are systematic reviews that summarize the technology standards and image technique for acquiring digital dermatological images [2]. In 1997, it was concluded that a resolution of 768x512 pixels would be adequate for the purposes of teledermatology [1, 3]. In 2008, for instance, practical guidelines from the American Telemedicine Association advised at least 24 color bits and in 2012 it recommended a resolution of 800x600 pixels, but preferably 1024x768 ones [1, 4, 10]. The standards of such techniques include ambient conditions (illumination, background, and camera position), patient pose, patient consent, privacy, and confidentiality [2].

The study of basic notions of photography is very important to the training of dermatologists. In Brazil, there are few training centers that offer it in their programs; however, this is already a subject much addressed in the national congresses, where minicourses take place for the dermatologists.

In the present study, sending the images by the application generated a resolution reduction of 3264x2448 to 1280x960 pixels, which is still within the established technology standards for the purposes of teledermatology, allowing, along with the standards of the technique, the analysis of an image quality.

We selected the WhatsApp to perform the mobile teledermoscopy after the verification that, with the sending of the image, the reduction of the resolution would not compromise the image analysis, since it still remained within the established technology standards for the purposes of teledermatology. The other reasons for choosing were the practicality offered by the application and the greater ease of replication.

What the studies clearly demonstrate is that even though the diagnostic accuracy of the lesions may be slightly lower for teledermatology, it has the ability to screen clearly benign lesions, allowing obvious malignancy and suspicious lesions to be properly managed in secondary care facilities [4]. We boldly emphasize, however, that, in order to achieve excellence in such a method, a rigorous protocol, including good clinical history, high-quality photography, and the safety of the dermatological examination, is pivotally necessary to recognize other suspicious lesions.

## 5. Conclusion 

Our study demonstrated a high concordance rate between the teledermoscopic and face-to-face diagnoses, comparable to those described in the medical literature, and it allows us to conclude that teledermoscopy, and in particular mobile teledermoscopy, is a promising method for the analysis of pigmented lesions and we firmly believe that it deserves attention for future applications, especially in our country, where the distribution of dermatologists is irregular and scarce in some regions, besides the nondominance of the dermatoscopic technique by a considerable portion of these ones. The latest medical census, for example, showed that the Southeast region concentrates 58.9% of the specialists, 15.8% throughout the South, 13.8% in the Northeast, 8% along the Midwest, and 3.5% throughout the North region. In absolute numbers, the variation ranges from 5 to 2183 dermatologists per state (Acre and São Paulo states, respectively) [1, 6].

We emphasize that the diagnosis did not influence the final decision making, which was based exclusively on face-to-face assessment, either in the diagnostic definition or in the established behavior; we also declare that there was no identification of the patients through the sent images, which contained only a numerical tag; we also affirm that the sharing of these ones was restricted to the two participating physicians; moreover, we emphasize that the data from anamnesis and physical examination are sometimes essential for this decision-making, especially when faced with doubtful lesions; we declare that the use of the dermoscopic adapter for image capture may have contributed positively to the quality of the images; and, lastly we affirm that the necessary infrastructure for the progress of this study was of minimal cost, with high practicality and functionality.

We conclude, hence, that prospective and randomized clinical studies, legal aspects, and systematized protocols are absolutely necessary for the advancement of mobile teledermoscopy in Brazil, once it stands out as a good diagnostic accuracy, practical, and low cost method.

## Figures and Tables

**Table 1:** Teledermoscopic and face-to-face diagnostics.

	Teledermoscopy	Face to face
Lesion 1	Pigmented basal cell carcinoma	Pigmented basal cell carcinoma
Lesion 2	Intradermal melanocytic nevus	Intradermal melanocytic nevus

Lesion 3	Intradermal melanocytic nevus	Intradermal melanocytic nevus
Lesion 4	Intradermal melanocytic nevus	Intradermal melanocytic nevus
Lesion 5	Intradermal melanocytic nevus	Intradermal melanocytic nevus
Lesion 6	Solar lentigo / seborrheic keratosis	Solar lentigo / seborrheic keratosis
Lesion 7	Benign melanocytic lesion	Benign melanocytic lesion
Lesion 8	Blue nevus	Blue nevus
Lesion 9	Intracorneal hematoma	Exogenous pigmentation
Lesion 10	Atypical melanocytic nevus	Atypical melanocytic nevus
Lesion 11	Intradermal nevus	Intradermal nevus
Lesion 12	Blue nevus	Blue nevus
Lesion 13	Seborrheic keratosis	Seborrheic keratosis
Lesion 14	Benign melanocytic lesion	Benign melanocytic lesion
Lesion 15	Benign melanocytic lesion	Benign melanocytic lesion
Lesion 16	Suspected melanocytic lesion	Benign melanocytic lesion
Lesion 17	Benign melanocytic lesion	Benign melanocytic lesion
Lesion 18	Pigmented basal cell carcinoma	Pigmented basal cell carcinoma
Lesion 19	Dermatofibroma	Dermatofibroma
Lesion 20	Intradermal nevus	Intradermal nevus
Lesion 21	Melanoma or atypical melanocytic nevus	Melanoma or atypical melanocytic nevus

Lesion 22	Seborrheic keratosis	Seborrheic keratosis
Lesion 23	Solar lentigo associated with blue nevus or melanocytic lesion with homogeneous eccentric pigmentation	Solar lentigo associated with blue nevus
Lesion 24	Benign melanocytic lesion	Benign melanocytic lesion
Lesion 25	Melanoma	Pigmented basal cell carcinoma
Lesion 26	Solar lentigo / seborrheic keratosis	Solar lentigo / seborrheic keratosis
Lesion 27	Congenital nevus	Congenital nevus
Lesion 28	Solar lentigo	Solar lentigo
Lesion 29	Melanoma	Melanoma
Lesion 30	Benign melanocytic lesion	Benign melanocytic lesion
Lesion 31	Benign melanocytic lesion	Benign melanocytic lesion
Lesion 32	Melanoma	Melanoma
Lesion 33	Solar lentigo	Solar lentigo
Lesion 34	Benign melanocytic lesion	Benign melanocytic lesion
Lesion 35	Pigmented basal cell carcinoma	Pigmented basal cell carcinoma
Lesion 36	Benign melanocytic lesion	Benign melanocytic lesion
Lesion 37	Benign melanocytic lesion	Benign melanocytic lesion
Lesion 38	Benign melanocytic lesion	Benign melanocytic lesion

Lesion 39	Suspected melanocytic lesion	Benign melanocytic lesion
Lesion 40	Lentigo maligna	Lentigo maligna

Lesion 41	Benign melanocytic nevus	Benign melanocytic nevus

## Data Availability

The data used to support the findings of this study are available from the corresponding author upon request.
